# Convolutional Neural Network-Based Finger-Vein Recognition Using NIR Image Sensors

**DOI:** 10.3390/s17061297

**Published:** 2017-06-06

**Authors:** Hyung Gil Hong, Min Beom Lee, Kang Ryoung Park

**Affiliations:** Division of Electronics and Electrical Engineering, Dongguk University, 30 Pildong-ro 1-gil, Jung-gu, Seoul 100-715, Korea; hell@dongguk.edu (H.G.H); mblee@dongguk.edu (M.B.L.)

**Keywords:** biometrics, finger-vein recognition, texture feature extraction, CNN

## Abstract

Conventional finger-vein recognition systems perform recognition based on the finger-vein lines extracted from the input images or image enhancement, and texture feature extraction from the finger-vein images. In these cases, however, the inaccurate detection of finger-vein lines lowers the recognition accuracy. In the case of texture feature extraction, the developer must experimentally decide on a form of the optimal filter for extraction considering the characteristics of the image database. To address this problem, this research proposes a finger-vein recognition method that is robust to various database types and environmental changes based on the convolutional neural network (CNN). In the experiments using the two finger-vein databases constructed in this research and the SDUMLA-HMT finger-vein database, which is an open database, the method proposed in this research showed a better performance compared to the conventional methods.

## 1. Introduction

Typical biometric technologies include face [1,2], fingerprint [3,4], iris [5,6], and finger-vein recognition [7,8]. In other studies [9], authors proposed 3D facial recognition based on 105 novel geometrical descriptors generated by composing primary geometrical descriptors such as mean, Gaussian, principal curvatures, shape index, curvedness, and the coefficients of the fundamental forms, and by applying standard functions such as sine, cosine, and logarithm to them. In other studies of 3D facial recognition [10], a method was proposed to automatically detect 11 landmarks from facial red-green-blue (RGB) images based on point-by-point mapping, of 11 differential geometry descriptors such as curvatures to the three individual RGB image components. In previous research [11], the performance comparisons of biometric technologies of face, fingerprint, iris, finger-vein, and voice are explained.

There are generally two factors that lower the recognition performance in finger-vein recognition systems: misalignment by translation and rotation of the finger, which occurs at the time of capturing the finger-vein image, and shading on the finger-vein image. The first factor involves the misalignment between the finger-vein patterns in the enrolled image and the recognition image due to the translation and rotation of the finger on the finger-vein image capturing device during a recognition attempt. The second factor involves a change in image quality due to shading that occurs in the input image, caused by the pressure of the finger touching the finger-vein image capturing device, because in general for finger-vein image capturing devices, the near-infrared (NIR) light emitting diode (LED) illuminates the finger from above or from the side.

To solve these problems, conventional finger-vein recognition algorithms perform the recognition based on the finger-vein lines extracted from the input images or image enhancement, and texture feature extraction [12] from the finger-vein images. In these cases, however, the inaccurate detection of finger-vein lines lowers the recognition accuracy. In the case of texture feature extraction, the developer must experimentally decide on a form of the optimal filter for extraction, considering the characteristics of image database. To address this problem, we propose a finger-vein recognition method that is robust to various database types and environmental changes (including misalignment and shading) based on the convolutional neural network (CNN). Our finger-vein recognition system can be used in various applications such as user authentication for a log-in system for a desktop computer, door access control, and driver verification [11].

In Section 2, the finger-vein recognition algorithms that have been researched to solve these problems are introduced.

## 2. Related Works

In general, a biometric system includes image acquisition, preprocessing, feature extraction, and matching. The finger-vein recognition system also has the same construction [13]. During preprocessing, the features of a finger-vein are extracted considering the specified region-of-interest (ROI), image resizing, image enhancement, and image alignment. The previous studies on finger-vein recognition mainly focused on preprocessing and feature extraction methods. In addition, some studies have applied Gabor filters of various directions and shapes to find the vein pattern [14,15,16,17,18,19,20,21,22]. Yang et al. proposed a method of finger-vein recognition, which involved extracting the features into 16 types of filters considering two scales, eight channels, and eight center frequencies of Gabor filters [15]. Peng et al. proposed a recognition method that is robust to scale and rotation, for which they designed an 8-way filter that selects the optimal parameters of the Gabor filter to extract the finger-vein features and applies the scale invariant feature transform (SIFT) algorithm to the features [18]. Yang et al. suggested a method of improving the contrast of the finger-vein pattern in the image using multi-channel even-symmetric Gabor filters with four directions [19]. Furthermore, in [20], they improved the quality of the finger-vein image by combining Gabor filtering with Retinex filtering based on a fuzzy inference system. In [21], they improved the quality of the finger-vein image through an optimal Gabor filter design based on the direction and thickness estimation of the finger-vein lines. Zhang et al. proposed gray-level grouping (GLG) in order to enhance the image contrast and a circular Gabor filter (CGF) to improve the quality of the finger-vein images [22]. In [23], both Gabor filter-based local features and global features of the moment-invariants method are used. In [24], they used an eight-channel Gabor filter to extract the features that were analyzed prior to the application of score-level fusion to obtain the final matching score.

Besides these Gabor filter methods, Pi et al. proposed a quality enhancement method for the finger-vein image based on edge-preserving and elliptical high-pass filters that can maintain the edges and remove the blurs [25]. In addition, Yu et al. suggested a multi-threshold method based on a fuzzy system considering the characteristics of the finger-vein pattern and the skin region [26]. Qian et al. [27] proposed a finger-vein recognition algorithm based on the fusion of score-level moment invariants by the weighted-average method.

Furthermore, studies were conducted using a local binary pattern (LBP) that considers the local patterns in various directions for finger-vein recognition [12,28]. Pham et al. enhanced the images of the vein using a Gabor filter and recognized the finger veins with the LBP algorithm. In addition, they analyzed the similarity and dissimilarity of the finger-vein patterns of the ten fingers [12]. In [28], Yang et al. used the binary features using the personalized best bit map (PBBM) extracted from the consistent bits identified in the LBP code for finger-vein matching. Later, the local line binary pattern (LLBP) method was proposed, which finds segments that are different from the local shapes of the neighbors in this LBP [13,29]. Line tracking methods to find the features of the blood vessels were also researched [30,31]. In previous research [7], they proposed the method of segmenting the finger area from the input image based on the gradient magnitude of spatial positions, and the method of extracting finger-vein lines based on the position-gray-profile curve. In another study [8], the authors proposed the finger-vein pattern-based human identification system where an image capturing device was made by a simple webcam and electronic circuit. In addition, the algorithms of finger-vein image denoising, binarization, and thinning were used for finger-vein recognition. Their methods showed good performance of extracting finger-vein patterns and recognition [7,8].

Furthermore, in other studies [32,33,34], the authors proposed a method of generating enhanced finger-vein images by considering the effect of the layered structure of skin and restoring the images based on a point-spread function (PSF) model [33], and a biological optical model (BOM) [34].

The improvement of recognition performance using other biometric data together with the finger-vein recognition system was researched for multimodal biometrics [27,35]. In [35], a finger-vein and finger geometry combined recognition system using a modified Gaussian high-pass filter through binarization, LBP, and local derivative pattern (LDP) was suggested. In [36], the authors proposed a method for combining the results of finger-vein and fingerprint recognitions using score level fusion [37].

Most of the existing studies on finger-vein recognition include non-training-based methods, which perform finger-vein recognition using various types of distance-based matchers that are based on the extracted finger-vein features. As such, it is difficult to provide excellent recognition performance for various kinds of finger-vein images obtained from diverse devices and environments. To solve this problem, the support vector machine (SVM) [38,39,40,41] and CNN [42,43] are being proposed, which are training-based methods. Among them, [40] researched, apart from finger-vein recognition, the presentation attack (spoof attack) detection to distinguish between the finger-vein of a living human being and a fake finger-vein printed or photographed with a smartphone. This research also used the SVM based on texture features extracted with steering pyramids for the final judgment. Such training-based methods have the advantage of being able to detect finger-vein patterns that are robust to various factors and environmental changes, because they reflect finger-vein images that have shading and misalignment together with the learning process.

Wu et al. proposed a finger-vein recognition method that applies the SVM and adaptive neuro-fuzzy inference system as classification methods after shortening the dimensions of the optimal features by applying the principal component analysis (PCA) and the linear discriminant analysis (LDA) to the finger-vein image [38]. Qin et al. proposed a classification method that applies the SVM after combining the vein shape, orientation, and scale-invariant feature transform (SIFT) features extracted from the finger-vein image [39]. Khellat-kihel et al. suggested a classification method using the SVM after enhancing the finger-vein image through a Gabor filter [41].

In addition, deep learning-based image recognition methods, which show high recognition performance through big data learning, are being applied to various fields, and as a part of this effort, finger-vein recognition using CNN has been researched. Radzi et al. proposed a method of using a reduced-complexity four-layer CNN with fused convolutional-subsampling architecture for finger-vein recognition [42]. Itqan et al. performed finger-vein recognition using CNN of the same structure as the one used in [42]. Whereas the CNN structure based on Linux MATLAB was used in [42], the CNN structure based on windows-based MATLAB was used in [43]. However, references [42,43] used finger-vein images of the same class both for training and testing, and for this reason, they cannot be applied to the finger-vein images of classes that have not been trained. This is because the CNN structure generally receives one image as input. In order to recognize finger-vein images of the untrained classes, a CNN structure that receives two images for authentic matching (matching between input and enrolled finger-vein images of the same class) or imposter matching (matching between input and enrolled finger-vein images of different classes) [44] that must be used, or a recognition method that includes separate distance matching with features in FCL in the step before the final fully connected layer (FCL) of the CNN obtained from one image must be used [45].

Considering the above-mentioned problems of the existing studies, we propose a finger-vein recognition method that is robust to various misalignments and shading and is based on the final FCL result of the CNN, without separate distant matching based on the features obtained from the CNN, with one image as the input. This research is novel in the following four ways compared to previous works.

To reduce the complexity of the conventional CNN structure that receives two images as the input, a method of obtaining one difference image from the two images for authentic matching or imposter matching and using this image as the CNN input is proposed.

In case that the features from the FCL of the step before the final FCL are used, a separate distance matching method must be used. However, in this case, the recognition performance is affected by the type of distance measuring method. Therefore, to solve this problem, instead of using the separate distance matching method based on the features, the authentic or imposter matching result is presented based on the final FCL result of the CNN.

Finger-vein recognition performance that is robust to various misalignments and shading was obtained by performing CNN training with three types of finger-vein image databases constructed in different environments. Furthermore, the two self-constructed databases in this research have been released through [46] for comparison of its performance with those in other studies.

The CNN structure that is appropriate for finger-vein recognition is analyzed by comparing the performance of the proposed CNN method with that of the existing various finger-vein recognition methods based on the non-trained method or various CNN structures.

Table 1 shows the comparisons of proposed and previous researches.

The composition of this paper is as follows. In Section 3, the structure and major parts of the proposed CNN-based finger-vein recognition system are described. In Section 4 and Section 5, the experimental results with analyses and the conclusion are provided.

## 3. Proposed Method

### 3.1. Overview of the Proposed System

**Figure 1:**
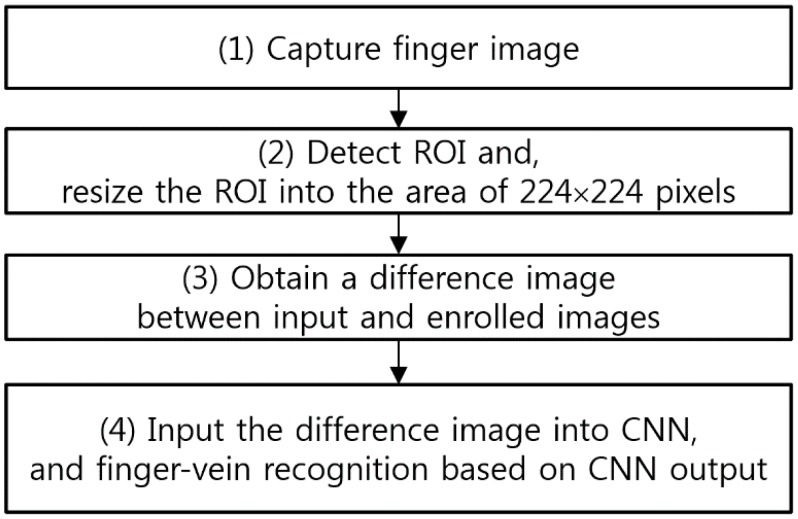
Flowchart of the proposed method.

Figure 1 shows the flowchart of the proposed finger-vein recognition method. The upper and lower boundaries of the finger are detected using two masks of 4 × 20 pixels from the images obtained from the finger-vein capturing device (step (1) of Figure 1), and the finger ROI is detected based on this (step (2) of Figure 1) [12]. The detected finger ROI is resized into an image of 224 × 224 pixels with no filtering or quality enhancement (step (2) of Figure 1), and then the difference image between the input and enrolled finger ROI image is obtained (step (3) of Figure 1). This difference image is used as the input to the pre-learned CNN, and the input finger-vein images are recognized based on the CNN output (step (4) of Figure 1).

**Figure 2:**
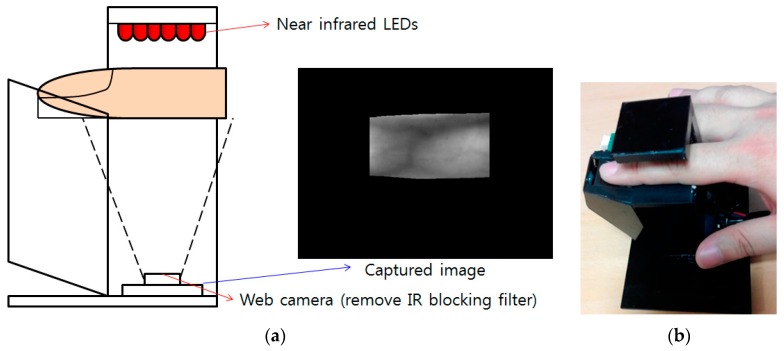
Finger-vein capturing device and its usage. (**a**) Finger-vein capturing device. (**b**) Example of using the device.

Figure 2 shows the finger-vein capturing device used to capture the finger-vein images for the database constructed in this research. This device consists of six 850 nm near-infrared (NIR) light emitting diodes (LEDs) [47] and a web camera (Logitech Webcam C600 [48]) as shown in Figure 2. The NIR cutting filter was removed from the web camera and an NIR passing filter [49] was added.

### 3.2. CNN-Based Finger-Vein Recognition

In this research, a test was conducted with a pre-trained model of VGG Net-16 [50] after fine-tuning this model with training images that consisted of difference images between the two images obtained from authentic matching or imposter matching. As explained in Section 4.3, besides the fine-tuning of the VGG Net-16, the testing performance was compared for various CNN structures, training methods, and types of inputs. Thus, VGG Net-16 fine tuning with the difference image as the input was used as the finger-vein recognition method based on recognition accuracy. A more detailed explanation about this is provided in Section 4.3.

**Figure 3:**
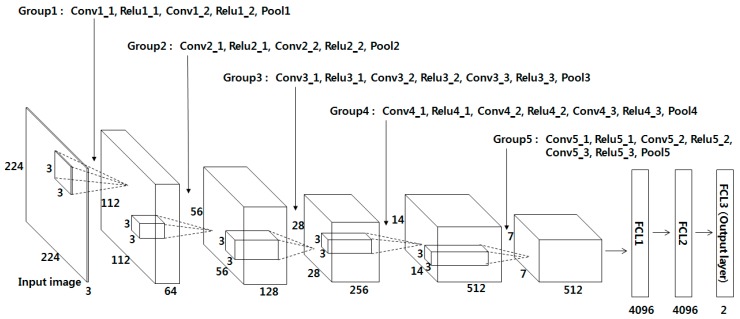
CNN architecture used in our research.

VGG Net-16 is composed of 13 convolutional layers, 5 pooling layers, and 3 FCLs, as shown in Table 2 and Figure 3. In the 1st convolutional layer, 64 filters of size 3 × 3 are used. Hence, the size of the feature map is 224 × 224 × 64 in the 1st convolutional layer, where 224 and 224 are the height and width of the feature map, respectively. They are calculated based on (output height (or width) = (input height (or width) – filter height (or width) + 2 × the number of padding)/the number of stride + 1 [51]). For example, in the image input layer and Conv1_1 in Table 2, the input height, filter height, number of padding, and number of strides are 224, 3, 1, and 1, respectively. As a result, the output height becomes 224 (= (224 − 3 + 2 × 1)/1 + 1). The rectified linear unit (Relu) layer can be expressed as follows [52,53,54]:

(1) y = max(0,x)

where x and y are the input and output values of the Relu function, respectively. The processing speed of the Relu function is usually faster than that of a non-linear activation function. This function can reduce the vanishing gradient problem [55] that can happen in cases when a hyperbolic tangent or sigmoid function is used in back-propagation for training. The feature map obtained by passing the Relu layer (Relu1_1) passes the 2nd convolutional layer and the Relu layer (Relu1_2) once again before passing the max pooling layer (Pool1), as shown in Table 2. As with the 1st convolutional layer, the filter size of 3 × 3, the padding of 1, and the stride of 0 are applied to the 2nd convolutional layer, and the 224 × 224 × 64 feature map size is maintained. As shown in Table 2, 13 convolutional layers maintain the same feature map size by using a filter size of 3 × 3 and padding of 1, and only the number of filters changes to 64, 128, 256, and 512. In addition, a Relu layer is connected to the back of each convolutional layer and the feature map size is maintained after passing the convolutional layer.

In the Max pooling layer, the maximum among the values of the defined filter range is used, which is a kind of subsampling. For example, Pool1 in Table 2 is the result of max pooling for the 2nd convolutional layer and Relu1_2. When the max-pooling layer (Pool1) is performed, the input feature map size is 224 × 224 × 64, the filter size is 2 × 2, and the number of strides is 2 × 2. Here, 2 × 2 for the number of strides implies the max pooling filter of 2 × 2 where the filter moves by two pixels in both the horizontal and vertical directions. As there is no overlapped area due to the filter movement, the feature map size diminishes to 1/4 (1/2 horizontally and 1/2 vertically). Consequently, the feature map size after passing Pool1 becomes 112 × 112 × 64 pixels. This pooling layer is used after Relu1_2, Relu2_2, Relu3_3, Relu4_3, and Relu5_3, as shown in Table 2. For all cases, the filter of 2 × 2 and stride of 2 × 2 are used and through this, the feature map size diminishes to 1/4 (1/2 horizontally and 1/2 vertically).

### 3.3. FCL of CNN

After the input image of 224 × 224 × 3 pixels passes the 13 convolutional layers, 13 Relu layers, and 5 pooling layers, a feature map of 7 × 7 × 512 pixels is finally obtained. In addition, it passes the three FCL layers. The numbers of output nodes of the 1st, 2nd, and 3rd FCLs are 4096, 4096, and 2, respectively. In this research, a verification structure was designed to determine whether the finger-vein image input by the CNN is the same vein image as the enrolled image (acceptance as genuine matching) or a different vein image (rejection as imposter matching). Finally, the 3rd FCL consists of two output nodes. For the 3rd FCL, the Softmax function is used, which can be expressed as follows [53]:

(2) $$\sigma {\left(p\right)}_{i}=\frac{e^{pi}}{\sum_{n=1}^{R}e^{pn}}$$

As shown in Equation (2), given that the array of output neurons is set as p, the probability of neurons corresponding to the ith class can be calculated by dividing the value of the ith element by the summation of the values of all the elements.

In general, CNN has an over-fitting problem, which can cause low recognition accuracy with the testing data although the accuracy with the training data is still high. To solve this problem, this research uses data augmentation and dropout methods [56,57], which can reduce the effects of the over-fitting problem. A detailed explanation about the experimental data generated by data augmentation is given in Section 4.1. For the dropout method, we adopt the dropout probability of 50% to disconnect the connections between the previous layer and the next layers in the FCL [56,57]. The dropout layer was used twice, that is, after the 1st FCL with Relu6 and after the 2nd FCL and Relu7, as shown in Table 2.

## 4. Experimental Results

### 4.1. Experimental Data and Environment

In this research, the training and testing were performed using three types of finger-vein databases. The first database was acquired with two guide bars (fingertip guide bar and finger side guide bar) attached, when the images were captured using the finger-vein capturing device shown in Figure 2 [12]. Ten images each were captured from the index, middle, and ring fingers of the left and right hands of 20 persons. Thus, this database has a total of 1200 images consisting of 20 people with 2 hands and 3 fingers, and 10 images per each finger. This is called the good-quality database, because the images have few finger-vein misalignments by finger translation due to the two guide bars, and there is little shading in the finger-vein images. For the second database, the two guide bars were removed when the images were captured with the device shown in Figure 2 [12]. Ten images each were captured from the index, middle, and ring fingers of the left and right hands of 33 persons. Thus, this database has a total of 1980 images consisting of 33 people with 2 hands and 3 fingers, and 10 images per each finger. This is called the mid-quality database because the images were captured with no guide bar, and the images have finger-vein misalignments by finger translation; however, there is little shading in the finger-vein images similar to the good-quality database. In this research, the good-quality database and the mid-quality database were disclosed for performance comparison by other researchers [46].

**Figure 4:**
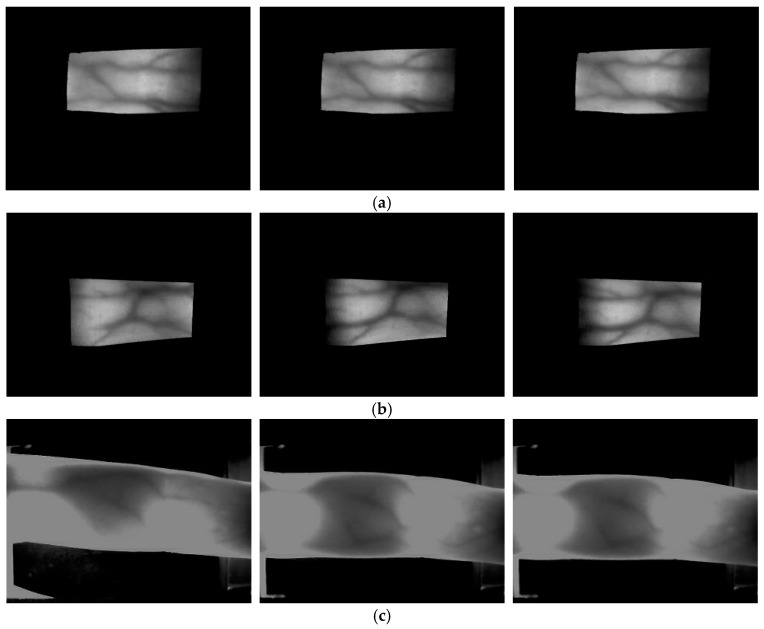
Examples of input images of different trials from the same finger of one individual from each database: (**a**) good-quality; (**b**) mid-quality; and (**c**) low-quality database.

The last database used in this research is the group of machine learning and applications, Shandong university-homologous multi-modal traits (SDUMLA-HMT) finger-vein database [58], for which six images each were captured from the index, middle, and ring fingers of both hands of 106 persons. A total of 3816 images were captured, which consists of 106 people with 2 hands and 3 fingers, and 6 images per each finger. The characteristics of the SDUMLA-HMT database are as follows. No guide bar was used when capturing the images as with the mid-quality database, and there are more finger-vein misalignments due to finger translation than in the good-quality and mid-quality databases. Furthermore, there is more shading in the finger-vein images than for the good-quality and mid-quality databases. Therefore, this is called the low-quality database as the image quality is the lowest among the three databases. Figure 4 shows examples of the good-quality, mid-quality, and low-quality databases used in this research.

The good-quality, mid-quality, and low-quality databases have 120 classes, 198 classes, and 636 classes, respectively. The classes from each database were divided into two groups, which were used for training and testing, respectively. In other words, in the good-quality database, images of 60 classes were used for training and the images of the remaining 60 classes were used for testing. In the mid-quality database, images of 99 classes were used for training and the images of the remaining 99 classes were used for testing. Finally, in the low-quality database, images of 318 classes were used for training and the images of the remaining 318 classes were used for testing. The mean accuracy was measured after two-fold cross validation by interchanging these images for training and testing.

Since the number of training data is insufficient for learning the many parameters and weights of 13 convolutional layers and 3 FCLs in the CNN shown in Table 2 and Figure 3, the number of training data was increased through data augmentation for this research. Table 3 shows the descriptions of the three databases and data augmentation used in this research.

In this research, the performance of the CNN-based finger-vein recognition was evaluated for two cases: using one original finger-vein image as the input to CNN during testing (case 1) and using one difference image obtained from the two finger-vein images of authentic matching (matching between input and enrolled finger-vein images of the same class) and imposter matching (matching between input and enrolled finger-vein images of different classes) (case 2). In case 1, where the classes used for training were different from those used for testing, the accuracy was measured through distance matching with enrolled features using the features of the immediately preceding FCL, instead of the final output values of the CNN. For example, in the case of the good-quality database in Table 3, the training was performed with 600 images of the 60 classes out of the total 120 classes and testing was performed with 600 images of the remaining 60 classes. In this case, the number of output nodes of CNN becomes 60 and CNN cannot be trained properly, because there are only 10 images for each class (over-fitting problem). To solve this problem, the number of training data was increased by data augmentation as described above. The popular image translation method was used for data augmentation [56]. In other words, the images were translated and cropped by 1 to 5 pixels in the up, down, left, and right directions, based on the coordinates of the original finger-vein image. In this way, 120 augmented images were obtained and 121 images from each original image were used for training. The number of augmented images in case 1 for each database is shown in Table 3.

In case 2, where one difference image obtained from two finger-vein images of authentic or imposter matching was used as the input to the CNN, the number of output nodes (output classes) of the CNN becomes two (class 1 (authentic matching) and class 2 (imposter matching)). For data augmentation, the number of images of each class was increased by 13 times using the aforementioned image translation method. In other words, the coordinates of the images were translated and cropped by 1 to 4 pixels in the up, down, left, and right directions, based on the coordinates of the original finger-vein image. As a result, 12 augmented images were obtained from one original image and 13 images for each class were used for training. For example, since the number of images for each class in Table 3 is 10 for the good-quality database, the number of images for each class becomes 130 (13 × 10) through this data augmentation. One of these 130 images was used for enrollment. Thus, the number of authentic matching becomes 129 cases for one image selected for the enrollment from each class. Consequently, the total authentic matching count becomes 7740 cases (129 cases/class × 60 classes). Since the imposter matching count is generally much greater than this, the same number of cases, that is 7740 cases, were randomly selected from the training data in order to avoid training over-fitting for the imposter matching. Thus, 7740 difference images were obtained from the authentic matching of 7740 cases, and 7740 difference images were obtained from the imposter matching of 7740 cases. Using this data does not cause training over-fitting in case 2, because the number of CNN output nodes is only two (two classes) in case 2, which is much smaller than that of case 1. Table 3 outlines the number of augmented images for case 2 in each database. The finger-vein misalignments between the enrolled image and the input image were compensated through the data augmentation based on the image translation in cases 1 and 2.

As described above, the augmented data was used for CNN training only, and the original images with no augmentation were used for the testing database to evaluate the performance. The performance of the testing database was also compared with respect to the two types of original vein images (case 1) and vein difference images (case 2). For a fair comparison, the numbers of authentic and imposter matching of case 1 were identical to those of case 2. In other words, the authentic matching count in the good-quality database was 540 (9 × 60), because, as shown in Table 3, there are 10 testing images for one class and 60 classes in total. In addition, the imposter matching count of the good-quality database becomes 31,860 (60 × 9 × 59). The authentic matching count of the mid-quality database was 891 (99 × 9), because there are 10 images in each class and there were 99 classes in total. Moreover, the imposter matching count of the mid-quality database becomes 87,318 (99 × 9 × 98). Finally, the authentic matching count of the low-quality database was 1590 (318 × 5), because there are six images in each class and there were 318 classes in total. Furthermore, the imposter matching count of the low-quality database was 504,030 (318 × 5 × 317).

In this research, the CNN training and testing was performed using a system with Intel® Core™ i7-6700 CPU @ 3.40 GHz (4 CPUs), 64 GB memory, and NVIDIA GeForce GTX TITAN X (3072 CUDA cores, NVIDIA Corporation, Santa Clara, U.S.) graphics card having a memory of 12 GB. The training and testing algorithms were implemented with Windows Caffe (version1) [59]. In addition, except for testing with the original finger-vein images (case 1), the testing performance was compared (case 3) even after performing the image enhancement by applying Gabor filtering to the original finger-vein image. In case 3, the image to which the same Gabor filtering [12] was applied was used for CNN training.

**Figure 5:**
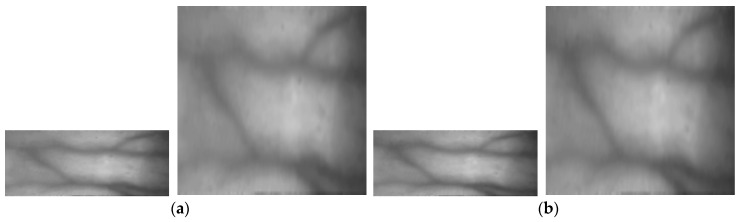

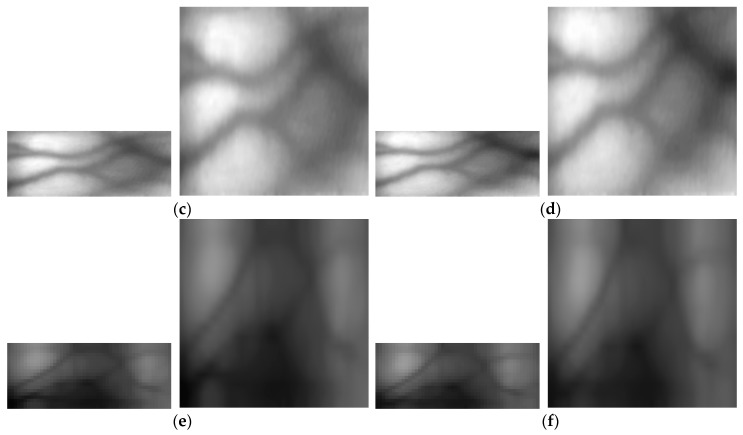
Examples of finger-vein ROI and their corresponding input image to the CNN (of the 1st (**a**,**c**,**e**) and 2nd trials (**b**,**d**,**f**) from the same finger of one individual) for case 1 training and testing: (**a**,**b**) good-quality; (**c**,**d**) mid-quality; and (**e**,**f**) low-quality database. In (**a**–**f**), the left and right images are the finger-vein ROI and its corresponding input image to the CNN, respectively.

Figure 5 shows examples of the finger-vein ROI and their corresponding input images to the CNN (of different trials from the same finger of one individual) for case 1 training and testing of the three databases.

As described above, the general CNN structure receives one image as the input [56]. Therefore, in order to recognize the finger-vein images of the non-trained classes, the CNN structure that receives two images of authentic matching (matching between input and enrolled finger-vein images of the same class) or imposter matching (matching between input and enrolled finger-vein images of different classes) as the input [44] must be used. Otherwise, the recognition method based on distance matching such as the cosine distance or Euclidian distance using the features of the FCL in the preceding step of the final FCL (Fc6 or Fc7 in Table 2) of CNN obtained from one image [37,45,60,61] must be used. In the case of the CNN structure that receives two images as the input, the complexity of the CNN structure and the difficulty of CNN training are much higher. When the features of Fc6 or Fc7 of the CNN obtained from one image are used, the distance matching must be used separately. Thus, in this research, one difference image was obtained from the two images of authentic matching or imposter matching and used as the CNN input (case 2 of Table 3).

### 4.2. Training of CNN

Table 4 lists the CNN models used in this research, which include a method of comparing the two images using the features in Fc7 with the original vein image as the input image (case 1) and a method of judging whether it is the same person or a different person by performing verification in the output layer (Fc8) after creating a difference image from the two images (case 2). For each case, a pre-trained model or a fine-tuned model was applied. For example, method A in Table 4 involves the comparison of two images by using the features in Fc7 with the original vein image as an input image in the VGG Face model (case 1). The VGG Face [62] model has the same CNN structure as that of VGG Net-16 [50] and the number of classes of the Fc8 output layer is 2622. Thus, this model was trained using approximately 2.6 million face images obtained from 2622 people.

Methods B and C compare the two images by using the features in Fc7 with the original vein images as the input to the VGG Net-16 and VGG Net-19 models, respectively (case 1). The VGG Net-16 and VGG Net-19 models [50] utilize 16 and 19 layers, respectively. As the number of classes of the output layer of Fc8 is 1000, this model was trained using approximately 1.3 million ImageNet Large-Scale Visual Recognition Challenge (ILSVRC) images. In these methods (A, B, and C), the performances of VGG Face, VGG Net-16, and VGG Net-19 models were evaluated with only the testing data and no additional training was performed with the vein images.

For the methods A-1, B-1, and C-1, fine-tuning was applied to the pre-trained models of VGG Face, VGG Net-16, and VGG Net-19, respectively. All the layers of the CNN were re-trained with the vein images, and the number of classes of the output layer of Fc8 varies depending on the database. In other words, the numbers of classes are 60, 99, and 318 for good-quality, mid-quality, and low-quality databases, respectively, as shown in Table 3.

Method D judges whether it is the same person or a different person by verifying the output layer (Fc8) after creating a difference image from the two images (case 2), using a structure with reduced numbers of filters and FCL nodes based on Alexnet [56]. Unlike the original Alexnet, which has 1000 classes of the output layer, two classes of the output layer were specified in this study, because verification was performed (authentic matching (class 1) and imposter matching (class 2)). For Method E, by reflecting the recent trend of small filter sizes such as 5 × 5 and 3 × 3 in Method D [50,62,63], the filter size was designed as 3 × 3, and the max-pooling count was adjusted from 3 to 5. Furthermore, the same feature output size of 2048 × 1 was applied to Fc6 and Fc7, and as with Method D, a verification structure was created with the two classes, authentic and imposter (case 2), using the difference image as the input, and then it was trained. Finally, for Method F, which is the method proposed in this research, the difference image was used as the input as well (case 2), and VGG Net-16 was fine-tuned with the vein image used in this research. The detailed structure is shown in Table 2 and Figure 3.

In this research, the stochastic gradient descent (SGD) method [64] was used for the CNN training. The SGD method finds the optimal weight that minimizes the difference between the desired output and the calculated output using the derivative. Unlike the Gradient descent (GD) method, in the SGD method, the training set divided by the mini-batch size is defined as the iteration, and the time when the training is completed for the iteration count is defined as one epoch. Then, training is performed for the predefined epoch count of 10.

**Figure 6:**
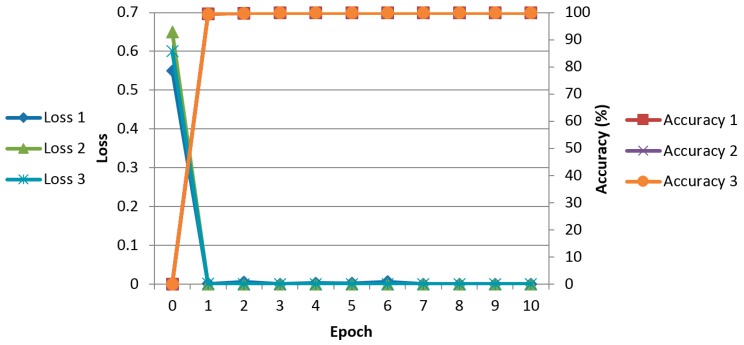
Examples of average loss and accuracy curves with the training data of two-fold cross validation according to the databases. The graphs of loss 1 (accuracy 1), loss 2 (accuracy 2), and loss 3 (accuracy 3) are obtained from good-, mid-, and low-quality databases, respectively.

Figure 6 shows the average graphs of loss and training accuracy of the two-fold cross validation when training is performed with Method F (proposed method) in Table 4. The X-axis represents the epoch count, the left Y-axis represents the loss value, and the right Y-axis represents the training accuracy. The loss value varies depending on the learning rate and batch size. If the learning rate is low, the loss value decreases gradually in a linear form. If the learning rate is high, the loss value decreases rapidly, but as the loss value varies rapidly, the loss value could stagnate without reaching the desired optimum training result. In this research, the optimum models, in which the loss curve converges close to 0 (0%) and the training accuracy converges close to 1 (100%), were chosen and used.

### 4.3. Testing of the Proposed CNN-Based Finger-Vein Recognition

#### 4.3.1. Comparative Experiments with the Original Finger-Vein Image and Gabor Filtered Image

As the first testing experiment, in order to find the vein images robust to finger-vein misalignment and shading when applied to the CNN, the finger-vein recognition performance was evaluated for two cases: when the original finger-vein image is used as the input to the CNN (case 1 in Table 3) and when the Gabor filtered image is used as the input to the CNN (case 3). For the CNN model, the recognition performance was evaluated using the B and B-1 methods in Table 4, although VGG Net-16 (fine-tuning) in Table 4 is used in this research, because one image is used as the input to the CNN. The performance was evaluated by two-fold cross validation. Using the 4096 features extracted from Fc7, the enrolled features based on the geometric center were specified, and the equal error rate (EER) of finger-vein recognition was measured by performing authentic and imposter matching based on the Euclidean distance with these enrolled features. The false rejection error rate (FRR) refers to the error rate of falsely rejecting the authentic matching attempt (recognizing an image of the same class as that of the enrolled finger-vein) as the imposter class. Conversely, the false acceptance error rate (FAR) refers to the error rate of falsely accepting (as enrolled finger-vein) an imposter matching attempt (recognizing an image of a class different from that of the enrolled finger vein) as a genuine class. In general, FRR and FAR are in a trade-off relation depending on the threshold for the finger-vein recognition. The error rate (FRR or FAR) is called EER in the case that FRR is equal to FAR [65,66].

**Figure 7:**
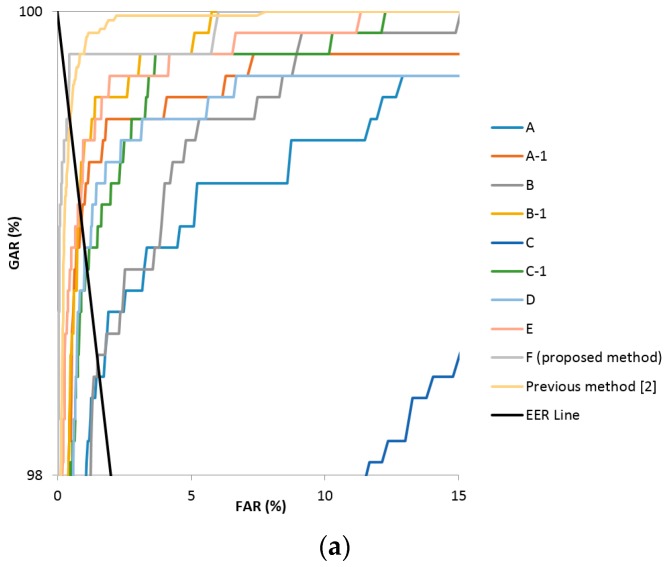

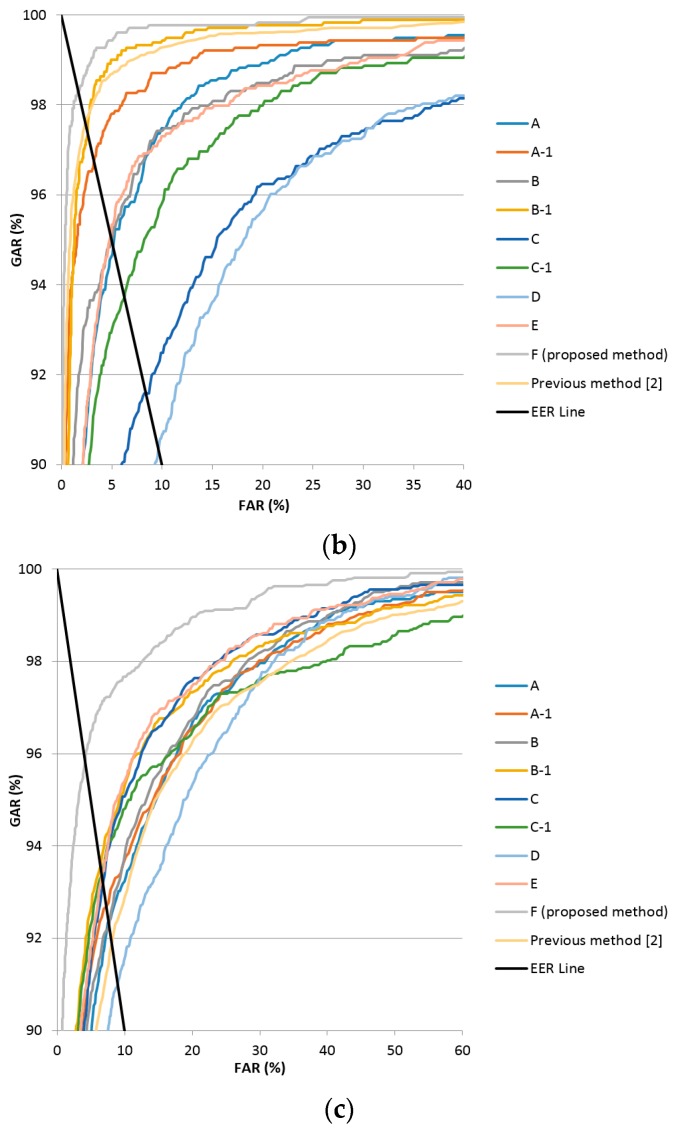
ROC curves of finger-vein recognition (**a**) good-quality, (**b**) mid-quality, (**c**) low-quality databases.

As shown in Table 5, when the results of applying Method B to the Gabor filtered image and the original image were compared, the EER using the Gabor filtered image was found to lower in the good-quality and mid-quality databases, whereas the EER using the original image was lower in the low-quality database. The reason for this is that, as shown in Figure 5 and Figure 7, in the case of the low-quality database, Gabor filtering could not obtain the effect of image enhancement, but noises increase, which affects the recognition, unlike the good-quality and mid-quality databases.

Furthermore, as shown in Table 5, the results of applying Method B-1 to the Gabor filtered image and the original image were compared. For the fine-tuning of VGG Net-16, the optimum fine-tuning model was experimentally found including the initial learning rates of 0.00001, 0.0005, and 0.0001. The momentum value was 0.9, and the size of the mini-batch was 20. The experimental results show that the application of fine-tuning to the original image produced a higher recognition accuracy compared to its application to the Gabor filtered image in all the databases. In addition, the recognition accuracy of the B-1 method with fine-tuning was higher, compared to the B method with no training. This result confirms that sufficient CNN training with only the original image, and without performing any image enhancement such as Gabor filtering, as preprocessing to the images, can produce a recognition performance that is robust to finger-vein misalignment and shading.

#### 4.3.2. Comparison of the Proposed Method with the Previous Method and Various CNN Nets

In the next experiment, the recognition accuracies of the various methods in Table 4, the proposed method, and the conventional method [12] were compared. The images used in this experiment were the original vein image (case 1 of Table 3) and the difference image (case 2 of Table 3). The testing performance was evaluated based on EER.

As shown in Table 6, for methods A, B, and C, the recognition accuracy was measured using the pre-trained model mentioned in Table 4 with no additional training with the finger-vein images. Thus, a lower EER than that for the previous method [12] can be obtained even with a pre-trained model unrelated to vein images in the case of the low-quality database. This is because, as shown in Figure 4 and Figure 5, in the case of the low-quality database, where the image quality including the visibility of the vein patterns is much lower than that of the good-quality and mid-quality databases, the valid features that can be used for recognition by LBP as in the previous method [12] were not detected, and more valid features were detected in the pre-trained CNN model, which is unrelated to the vein images. Furthermore, even the pre-trained CNN model that is unrelated to the vein image is more robust to finger-vein misalignment and shading than the LBP is.

A-1, B-1, and C-1 in Table 6 show the testing EER measured by fine-tuning the CNN models of methods A, B, and C with the finger-vein training dataset used in this research. The methods A-1, B-1, and C-1 produced lower EERs in all the three types of databases compared to the conventional methods A, B, and C, which are unrelated to the vein images as shown in Table 6.

Method D, using the vein difference image, reduced the filter count and the FCL weight value from the existing Alexnet as described in Table 4. It showed a high EER as a result of using larger sized filters of 11 × 11 pixels, because the blood vessel data of the veins were lost in the process of passing multiple convolutional layers. Method E, which uses a smaller filter to solve this problem (see Table 4), showed generally lower EERs compared to method D, but still did not produce better performance results in the good-quality and mid-quality databases compared to the previous method [12]. In the final analysis, finger-vein recognition using method F, which fine-tuned the VGG Net-16 model with the finger-vein difference image, was found to produce the lowest EER in this research. Figure 7 shows the receiver operating characteristic (ROC) curves of finger-vein recognition for the proposed methods and previous research [12] in Table 6. The horizontal and vertical axes of each graph represent FAR and the genuine acceptance error rate (GAR), respectively. GAR is calculated by the formula 100 − FRR (%). As explained in Section 4.3.1, FRR decreases (GAR increases) according to the increase of FAR by the change of the threshold for the finger-vein recognition. The ROC curve shows the graph of this increase of GAR according to the increase of FAR. The method of having higher GAR than others at the same FAR is regarded as showing higher accuracy of finger-vein recognition. For example with Figure 7c, at the FAR of 10%, the GAR of F (proposed method) is about 97.7% which is higher than those of all the other methods (the second highest GAR is about 95.4% by the E method). Therefore, the closer to the left-top position (FAR of 0% and GAR of 100%) the ROC curve is, the higher the accuracy of the method is compared to others. Because the ROC curves of F (proposed method) are closer to the left-top position than other methods in Figure 7a–c, we can confirm that the finger-vein recognition accuracy of the proposed method is highest among the various methods with all three types of databases. In addition, the black sold line represents the EER line where FAR is the same as FRR.

The inaccurate detection of finger-vein lines lowers the recognition accuracy. In the case of texture feature extraction, the developer must experimentally decide on a form of the optimal filter for extraction considering the characteristics of the image database. Different from the others, the CNN-based method uses the input image (without the detection of the finger-vein line), and all the optimal filters with optimal classifiers can be automatically determined by the training procedure (without the manual selection of filters and classifiers). Through intensive training with various databases, its performance can become robust to various database types and environmental changes (including misalignment and shading). These are the important features and merits of the CNN-based method. However, the intensive training usually takes a long processing time with a lot of training data. In the actual experimental environment, it is often the case to have difficulty in collecting lots of training data, and data augmentation is performed, consequently. This is the demerit of the CNN-based method, and it is the obstacle for the implementation of CNN in real practice. To reduce this demerit and obstacle, we make our trained CNN model for finger-vein recognition with collected databases open to other researchers through [46]. Therefore, they can easily use our system, and compare the performance with our CNN model and databases.

## 5. Conclusions

A CNN-based finger-vein recognition that is robust to finger-vein misalignment and shading was researched using images captured with a vein recognition system. The CNN was trained to make it robust to various environmental changes using three types of databases with various characteristics obtained from the camera of the vein recognition system. The recognition performances of various CNN models were compared by using enhanced images, original images with no preprocessing, and difference images obtained from two finger-vein images for CNN training. The experimental results showed that the fine-tuning of VGG Net-16 based on difference images, which is proposed in this research, achieved higher recognition accuracy compared to the existing method and various other CNN structures, in all three types of databases.

By using one difference image as the input to CNN, the complicated CNN structure considering two inputs is not necessary to be used in our research. In addition, the further procedure of distance measuring based on the extracted features from CNN FCL is unnecessary. Instead, the final decision of genuine and imposter matching in finger-vein recognition can be made based on the output of CNN. In order to successfully train the proposed deep CNN model, lots of training data are required. However, in many experimental environments, it is often the case to have difficulty in collecting this amount of data. Therefore, the increment of training data by correct data augmentation is necessary, which should reflect the characteristics of the original training data. To reduce this demerit and obstacle of the CNN-based method, we made our trained CNN model for finger-vein recognition with collected databases open to other researchers through [46].

In our future work, the proposed CNN method will be applied to other types of vein images (palm-vein, hand-vein etc.) and their performances will be evaluated. In addition, we will conduct research on the combination of different biometric data with the CNN method proposed in this research in a multi-modal way.

## Figures and Tables

**Table 1:** Comparisons of proposed and previous studies on finger-vein recognition.

Category		Methods	Strength		Weakness
Non-training-based	Image enhancement based on the blood vessel direction	Gabor filter [14,15,16,17,18,19,20,21,22], Edge-preserving and elliptical high-pass filters [25]	Improved finger-vein recognition accuracy based on clear quality images		Recognition performance is affected by the misalignment and shading of finger-vein images.
	Method considering local patterns of blood vessel	Local binary pattern (LBP) [12], personalized best bit map (PBBM) [28]	Processing speed is fast because the entire texture data of ROI is used without detecting the vein line		
	Method considering the vein line characteristics	LLBP [13,29]	Recognition accuracy is high because the blood vessel features are used instead of the entire texture data of ROI		
		Vein line tracking [30,31]			
Training-based		SVM [38,39,40,41]	Robust to various factors and environmental changes because many images with shading and misalignments are learned.		A separate process of optimal feature extraction and dimension reduction is required for the input to SVM
		CNN	Reduced-complexity four-layer CNN [42,43]	A separate process of optimal feature extraction and dimension reduction is not necessary	Cannot be applied to finger-vein images of non-trained classes
			Proposed method	Finger-vein images of non-trained classes can be recognized	The CNN structure is more complex than existing methods [42,43]

**Table 2:** The proposed CNN configuration used in our research.

Layer Type		Number of Filter	Size of Feature Map	Size of Kernel	Number of Stride	Number of Padding
Image input layer			224 (height) × 224 (width) × 3 (channel)			
Group 1	Conv1_1 (1st convolutional layer)	64	224 × 224 × 64	3 × 3	1 × 1	1 × 1
	Relu1_1		224 × 224 × 64			
	Conv1_2 (2nd convolutional layer)	64	224 × 224 × 64	3 × 3	1 × 1	1 × 1
	Relu1_2		224 × 224 × 64			
	Pool1	1	112 × 112 × 64	2 × 2	2 × 2	0 × 0
Group 2	Conv2_1 (3rd convolutional layer)	128	112 × 112 × 128	3 × 3	1 × 1	1 × 1
	Relu2_1		112 × 112 × 128			
	Conv2_2 (4th convolutional layer)	128	112 × 112 × 128	3 × 3	1 × 1	1 × 1
	Relu2_2		112 × 112 × 128			
	Pool2	1	56 × 56 × 128	2 × 2	2 × 2	0 × 0
Group 3	Conv3_1 (5th convolutional layer)	256	56 × 56 × 256	3 × 3	1 × 1	1 × 1
	Relu3_1		56 × 56 × 256			
	Conv3_2 (6th convolutional layer)	256	56 × 56 × 256	3 × 3	1×1	1 × 1
	Relu3_2		56 × 56 × 256			
	Conv3_3 (7th convolutional layer)	256	56 × 56 × 256	3 × 3	1 × 1	1 × 1
	Relu3_3		56 × 56 × 256			
	Pool3	1	28 × 28 × 256	2 × 2	2 × 2	0 × 0
Group 4	Conv4_1 (8th convolutional layer)	512	28 × 28 × 512	3 × 3	1 × 1	1 × 1
	Relu4_1		28 × 28 × 512			
	Conv4_2 (9th convolutional layer)	512	28 × 28 × 512	3 × 3	1 × 1	1 × 1
	Relu4_2		28 × 28 × 512			
	Conv4_3 (10th convolutional layer)	512	28 × 28 × 512	3 × 3	1 × 1	1 × 1
	Relu4_3		28 × 28 × 512			
	Pool4	1	14 × 14 × 512	2 × 2	2 × 2	0 × 0
Group 5	Conv5_1 (11th convolutional layer)	512	14 × 14 × 512	3 × 3	1 × 1	1 × 1
	Relu5_1		14 × 14 × 512			
	Conv5_2 (12th convolutional layer)	512	14 × 14 × 512	3 × 3	1 × 1	1 × 1
	Relu5_2		14 × 14 × 512			
	Conv5_3 (13th convolutional layer)	512	14 × 14 × 512	3 × 3	1 × 1	1 × 1
	Relu5_3		14 × 14 × 512			
	Pool5	1	7 × 7 × 512	2 × 2	2 × 2	0 × 0
Fc6 (1st FCL)			4096 × 1			
Relu6			4096 × 1			
Dropout6			4096 × 1			
Fc7 (2nd FCL)			4096 × 1			
Relu7			4096 × 1			
Dropout7			4096 × 1			
Fc8 (3rd FCL)			2 × 1			
Softmax layer			2 × 1			
Output layer			2 × 1			

**Table 3:** Descriptions of the three databases used in our research (*: index, middle, and ring fingers, **: from authentic matching, ***: from imposter matching).

			Good-Quality Database	Mid-Quality Database	Low-Quality Database
Original images		# of images	1200	1980	3816
		# of people	20	33	106
		# of hands	2	2	2
		# of fingers *	3	3	3
		# of classes (# of images per class)	120 (10)	198 (10)	636 (6)
Data augmentation for training	CNN using original image as input (Case 1)	# of images	72,600 (60 classes × 10 images × 121 times)	119,790 (99 classes × 10 images × 121 times)	230,868 (318 classes × 6 images × 121 times)
	CNN using difference image as input (Case 2)	# of images	15,480	25,542	48,972
			7740 ** ((10 images × 13 times – 1) × 60 classes)	12,771 ** ((10 images × 13 times – 1) × 99 classes)	24,486 ** ((6 images × 13 times – 1) × 318 classes)
			7740 ***	12,771 ***	24,486 ***

**Table 4:** Various CNN models for comparisons (convN means the filter of N×. For example, conv3 represents the filter of 3 × 3).

Input Image	Original Image as Input to CNN (Case 1 of Table 2)			Difference Image as Input to CNN (Case 2 of Table 2)		
Net configuration	VGG Face (no fine-tuning/fine-tuning)	VGG Net-16 (no fine-tuning/fine-tuning)	VGG Net-19 (no fine-tuning/fine-tuning)	Revised Alexnet-1 (whole training)	Revised Alexnet-2 (whole training)	VGG Net-16 (fine-tuning) (Proposed method)
Method name	A/A−1	B/B−1	C/C−1	D	E	F
# of layers	16	16	19	8	8	16
Filter size (# of filters)	conv3 (64) conv3 (64)	conv3 (64) conv3 (64)	conv3 (64) conv3 (64)	Conv11 (96)	conv3 (64)	conv3 (64) conv3 (64)
Pooling type	MAX	MAX	MAX	MAX	MAX	MAX
Filter size (# of filters)	conv3 (128) conv3 (128)	conv3 (128) conv3 (128)	conv3 (128) conv3 (128)	Conv5 (128)	conv3 (128)	conv3 (128) conv3 (128)
Pooling type	MAX	MAX	MAX	MAX	MAX	MAX
Filter size (# of filters)	conv3 (256) conv3 (256) conv3 (256)	conv3 (256) conv3 (256) conv3 (256)	conv3 (256) conv3 (256) conv3 (256) conv3 (256)	conv3 (256)	conv3 (256)	conv3 (256) conv3 (256) conv3 (256)
Pooling type	MAX	MAX	MAX		MAX	MAX
Filter size (# of filters)	conv3 (512) conv3 (512) conv3 (512)	conv3 (512) conv3 (512) conv3 (512)	conv3 (512) conv3 (512) conv3 (512) conv3 (512)	conv3 (256)	conv3 (256)	conv3 (512) conv3 (512) conv3 (512)
Pooling type	MAX	MAX	MAX		MAX	MAX
Filter size (# of filters)	conv3 (512) conv3 (512) conv3 (512)	conv3 (512) conv3 (512) conv3 (512)	conv3 (512) conv3 (512) conv3 (512) conv3 (512)	conv3 (128)	conv3 (128)	conv3 (512) conv3 (512) conv3 (512)
Pooling type	MAX	MAX	MAX	MAX	MAX	MAX
Fc6 (1st FCL)	4096	4096	4096	4096	2048	4096
Fc7 (2nd FCL)	4096	4096	4096	1024	2048	4096
Fc8 (3rd FCL)	2622/# of class	1000/# of class	1000/# of class	2	2	2

**Table 5:** Comparison of the error rates of finger-vein recognition between the original vein image (case 1 in Table 3) and the Gabor filtered image (case 3) in VGG Net-16.

Method Name	Input Image	EER (%)		
		Good-Quality Database	Mid-Quality Database	Low-Quality Database
B (of Table 4) (VGG Net-16 (no fine-tuning))	Gabor filtered image	1.078	4.016	7.905
	Original image	1.481	4.928	7.278
B-1 (of Table 4) (VGG Net-16 (fine-tuning))	Gabor filtered image	0.830	3.412	7.437
	Original image	0.804	2.967	6.115

**Table 6:** Comparison of recognition accuracy among the previous method, various CNN nets, and the proposed method.

Method Name	Input Image	Features (or Values) Used for Recognition	EER (%)		
			Good-Quality Database	Mid-Quality Database	Low-Quality Database
Previous method [12]	Original image	-	0.474	2.393	8.096
A (VGG Face (no fine-tuning))		Fc7	1.536	5.177	7.264
A-1 (VGG Face (fine-tuning))		Fc7	0.858	3.214	7.044
B (VGG Net-16 (no fine-tuning))		Fc7	1.481	4.928	7.278
B-1 (VGG Net-16 (fine-tuning))		Fc7	0.804	2.967	6.115
C (VGG Net-19 (no fine-tuning))		Fc7	4.001	8.216	6.692
C-1 (VGG Net-19 (fine-tuning))		Fc7	1.061	6.172	6.443
D (Revised Alexnet-1 (whole training))	Difference image	Fc8	0.901	8.436	8.727
E (Revised Alexnet-2 (whole training))		Fc8	0.763	4.767	6.540
F (VGG Net-16 (fine-tuning) **(proposed method)**)		Fc8	0.396	1.275	3.906
